# BPS and BPF linked to metabolic alterations independent of adiposity in Thai children and adolescents

**DOI:** 10.1265/ehpm.26-00027

**Published:** 2026-06-04

**Authors:** Watcharin Netiwuttisan, Ekkachai Nakaviroj, Nattakarn Numsriskulrat, Pajaree Chariyavilaskul, Patthamapon Adchariyasakulchai, Suphab Aroonparkmongkol, Khomsak Srilanchakon, Vichit Supornsilchai

**Affiliations:** 1 Division of Pediatric Endocrinology, Department of Pediatrics, Faculty of Medicine, Chulalongkorn University, Bangkok, Thailand; 2 Academic Services Affairs and International Education, Faculty of Medicine, Chulalongkorn University, Bangkok, Thailand; 3 Center of Excellence in Clinical Pharmacokinetics and Pharmacogenomics (PKPGx), Faculty of Medicine, Chulalongkorn University, Bangkok, Thailand; 4 Pharmacogenomics Laboratory, Center for Medical Diagnostic Laboratories, Faculty of Medicine, Chulalongkorn University, Bangkok, Thailand; 5 Department of Pharmacology, Faculty of Medicine, Chulalongkorn University, Bangkok, Thailand

**Keywords:** Bisphenol A, Bisphenol S, Bisphenol F, Childhood obesity, Insulin resistance, Hyperinsulinemia, Metabolic disorders

## Abstract

**Background:**

Bisphenol A (BPA) and its structural substitutes, bisphenol S (BPS) and bisphenol F (BPF), are widely used endocrine-disrupting chemicals. While BPA exposure has been linked to metabolic disorders, evidence regarding BPA substitutes in children remains limited, particularly in Southeast Asia.

**Methods:**

We conducted a cross-sectional study among Thai children and adolescents aged 7–18 years classified as normal weight (NW) or overweight/obese (OW/OB). Urinary BPA, BPS, and BPF concentrations were measured using liquid chromatography–tandem mass spectrometry. Anthropometric measurements, body composition, fasting glucose, insulin, lipid profiles, and liver enzymes were assessed. Associations between urinary bisphenol concentrations and metabolic parameters were analyzed using multivariable regression models.

**Results:**

A total of 114 participants were included (26 normal weight and 88 overweight/obese). BPA was detected in all (100%) urine samples, whereas BPS and BPF were detected in 50.0% and 53.6% of participants, respectively. Creatinine-adjusted urinary concentrations of BPA, BPS, and BPF did not differ significantly between NW vs. OW/OB. Higher urinary BPS and BPF concentrations were associated with higher fasting insulin levels (aGMR 1.13; 95%CI 1.05–1.20; p = 0.001) and higher LDL cholesterol levels (aGMR 5.82; 95%CI 0.52–11.11; p = 0.032), respectively. In contrast, higher urinary BPA concentrations were associated with lower fasting insulin levels (aGMR 0.77; 95%CI 0.66–0.90; p = 0.001).

**Conclusions:**

Exposure to BPA and its substitutes, BPS and BPF, were associated with distinct metabolic alterations independent of weight status. BPS was associated with hyperinsulinemia, while BPF was associated with elevated LDL cholesterol.

**Supplementary information:**

The online version contains supplementary material available at https://doi.org/10.1265/ehpm.26-00027.

## Introduction

The prevalence of childhood obesity in Thailand has more than doubled over the past two decades and represents a major public health concern. According to national surveillance data, the prevalence of obesity among children aged 2–5 years increased from 5.8% in 1997 to 7.9% in 2001, while that among children aged 6–12 years increased from 5.8% to 6.7% [1]. Childhood obesity is associated with a wide range of adverse health outcomes, including insulin resistance, dyslipidemia, metabolic dysfunction-associated steatotic liver disease (MASLD), and an increased risk of metabolic syndrome later in life.

In addition to lifestyle factors such as dietary habits and physical inactivity, increasing evidence suggests that exposure to endocrine-disrupting chemicals (EDCs) may contribute to the development of obesity and metabolic disorders [2, 3]. Bisphenol A (BPA) is one of the most widely studied EDCs and has been extensively used in the production of polycarbonate plastics, epoxy resins, food containers, and thermal paper. BPA can interact with estrogen, androgen, and thyroid hormone receptors, thereby affecting metabolic regulation, adipogenesis, and glucose homeostasis [2, 4].

Due to regulatory restrictions on BPA use in many countries, structural analogues such as bisphenol S (BPS) and bisphenol F (BPF) have increasingly been introduced as substitutes [5, 6]. Emerging experimental and epidemiological data suggest that these BPA analogues may exert endocrine-disrupting effects comparable to or even exceeding those of BPA [6–9]. However, data regarding their metabolic effects, particularly in pediatric populations, remain limited. Several studies have reported positive associations between urinary BPA concentrations and overweight or obesity in children [6, 10–14]. Meta-analyses have demonstrated that higher BPA exposure is associated with increased body mass index (BMI), waist circumference, and risk of obesity [11]. In contrast, evidence linking BPA exposure to components of metabolic syndrome and body composition in children has been inconsistent [15–23]. Furthermore, studies examining the associations between BPA analogues (BPS and BPF) and metabolic outcomes in children are scarce, and data from Southeast Asian populations are particularly limited [24].

Given the rising prevalence of childhood obesity in Thailand and the increasing exposure to BPA substitutes, there is a critical need to elucidate the metabolic effects of bisphenol analogues in children and adolescents. Therefore, this study aimed to investigate the associations between urinary concentrations of BPA, BPS, and BPF and (1) overweight/obesity and (2) obesity-related metabolic abnormalities, including insulin resistance, dyslipidemia, and liver enzyme abnormalities, in Thai children and adolescents.

## Methods

### Study design and participants

This cross-sectional study was conducted at King Chulalongkorn Memorial Hospital, Bangkok, Thailand, between July 1, 2024, and January 31, 2025. Children and adolescents aged 7–18 years who attended the pediatric outpatient or inpatient clinics, as well as those who voluntarily participated in the study, were eligible for enrollment.

Participants were classified into two groups based on BMI z-scores according to World Health Organization reference standards. The overweight/obese (OW/OB) group included participants with a BMI z-score >+1 standard deviation (SD) (overweight) and >+2 SD (obesity). The normal-weight (NW) group included participants with a BMI z-score between −1 and +1 SD. Exclusion criteria were secondary causes of obesity, use of systemic corticosteroids, and underlying chronic illnesses that could affect growth or metabolic parameters.

### Data collection and anthropometric measurements

Baseline demographic data, including age, sex, nationality, underlying medical conditions, caregiver socioeconomic characteristics, and potential sources of bisphenol exposure, were collected using standardized questionnaires. Body weight and height were measured using calibrated instruments, and BMI was calculated as weight (kg) divided by height squared (m^2^). BMI z-scores were determined using age- and sex-specific reference values. Pubertal status was assessed using a self-administered questionnaire based on Tanner staging.

### Body composition assessment

Body composition was assessed using bioelectrical impedance analysis (InBody 770; InBody Co., Seoul, Korea). Participants stood upright on the device with both feet placed on the electrodes and held the hand electrodes according to the manufacturer’s instructions. The device automatically calculated body fat mass, skeletal muscle mass, percentage body fat, and segmental fat distribution.

### Urine sample collection and bisphenol analysis

Spot urine samples (10–20 mL) were collected in bisphenol A–free containers and stored according to standardized procedures. Urinary concentrations of BPA, BPS, and BPF were measured using liquid chromatography–tandem mass spectrometry (LC–MS/MS; SHIMADZU LCMS-8060, Kyoto, Japan). Urine samples (1 mL) were centrifuged at 3,000 rpm for 15 minutes at room temperature. A 500-µL aliquot of the supernatant was incubated with ammonium acetate (10 µL) and β-glucuronidase (20 µL) at 37 °C for 3 hours. Acetonitrile (500 µL) and internal standards (d16-BPA, d8-BPS, and d10-BPF; 8 µL) were then added, followed by centrifugation at 3,000 rpm for 15 minutes. The supernatant was filtered through a 0.22-µm nylon syringe filter prior to LC–MS/MS analysis. LOQ (Limit of Quantification) is the lowest concentration or amount of an analyte in a sample that can be quantitatively determined with acceptable accuracy and precision under the stated experimental conditions. The LOQ for BPA, BPF, and BPS were 0.50, 0.01, and 0.01 ng/mL, respectively. Urinary creatinine concentrations were measured to adjust bisphenol concentrations for urine dilution. The linear calibration curve (r^2^ ≥ 0.99) of BPF and BPS was 0.01–100 ng/ul (0.01, 0.5, 1, 5, 10, 20, 50, 100 ng/ml), and 0.5–100 ng/ul (0.5, 1, 5, 10, 20, 50, 100 ng/ml) for BPA. For method validation, all samples were subtracted with blank matrix before analysis and all laboratory consumables used were bisphenol-free and decontaminated by washing with methanol. The accuracy and precision of the method were determined using intra-day and inter-day assays (n = 5) at each quality control (QC) level (Supplement Table 1). Additionally, the extraction recovery was evaluated to ensure the efficiency of the sample preparation process. The recoveries of bisphenol compounds were 88.75–112.02% (Supplement Table 2). The matrix effects in urine were 92.73–113.33 (4.56–18.99% CV).

### Blood sample collection and laboratory analysis

After an overnight fast of at least 6–8 hours, venous blood samples were collected to measure fasting plasma glucose (FPG), glycated hemoglobin (HbA1c), fasting insulin, lipid profiles; total cholesterol, triglycerides (TG), low-density lipoprotein cholesterol (LDL), and high-density lipoprotein cholesterol (HDL), as well as liver enzymes including aspartate aminotransferase (AST) and alanine aminotransferase (ALT). Insulin resistance and insulin sensitivity were estimated using the homeostasis model assessment indices; Homeostatic Model Assessment of Insulin Resistance (HOMA-IR), Insulin Sensitivity (HOMA-IS).

### Definitions

Diabetes mellitus was defined according to established diagnostic criteria, including fasting plasma glucose ≥ 126 mg/dL, 2-hour plasma glucose ≥ 200 mg/dL following a 75-g oral glucose tolerance test, HbA1c ≥ 6.5%, or the presence of classic symptoms of hyperglycemia with a random plasma glucose ≥ 200 mg/dL.

Metabolic syndrome was defined according to age-specific criteria. For children aged 6–10 years, metabolic syndrome was not formally diagnosed but evaluated in the presence of a family history of type 2 diabetes mellitus, dyslipidemia, cardiovascular disease, or hypertension. For participants aged 10–16 years, metabolic syndrome was defined as central obesity plus at least two of the following: TG ≥ 150 mg/dL, HDL cholesterol ≤ 40 mg/dL, systolic blood pressure ≥ 130 mmHg or diastolic blood pressure ≥ 85 mmHg, or FPG ≥ 100 mg/dL. Participants older than 16 years were assessed using adult diagnostic criteria.

### Statistical analysis

Continuous variables are presented as mean ± standard deviation (SD) or median (interquartile range), as appropriate. Categorical variables are presented as frequencies and percentages. Comparisons between groups were performed using the chi-square test or Fisher’s exact test for categorical variables and the unpaired Student’s t-test or Mann–Whitney U test for continuous variables, depending on data distribution. Urinary bisphenol concentrations and metabolic parameters were natural log-transformed prior to analysis. Associations between urinary bisphenol concentrations and metabolic parameters were analyzed using geometric mean ratios (GMRs) with 95% confidence intervals. Bisphenol concentrations below the LOQ were calculated as LOQ/ √2 for statistical analyses [25]. Variables exhibiting a p-value of less than 0.20 in the univariable analysis, along with the bisphenol concentration and those identified as influential factors in existing literature, such as age (years), obesity status, BMI SDS, body fat mass (kg), SMM (kg), PBF (%), pubertal status, fasting plasma glucose (mg/dL), Insulin (µU/mL), HOMA IR, ALT (IU/L) and LDL (mg/dL), were entered into the multivariable regression model. To identify independent predictors and control for confounding, a backward stepwise elimination procedure was applied. During this process, we assessed and addressed multicollinearity between covariates to ensure the stability of the final model. A two-sided p-value < 0.05 was considered statistically significant. All statistical analyses were performed using STATA19 (StataCorp LLC, College Station, TX).

### Ethical approval

The study protocol was approved by the Institutional Review Board of the Faculty of Medicine, Chulalongkorn University (IRB 0183/67). Written informed consent was obtained from parents or legal guardians, and assent was obtained from participants when appropriate.

## Results

### Participant characteristics

Between July 1, 2024, and January 31, 2025, a total of 117 participants were recruited. Three participants were excluded due to incomplete data, resulting in 114 participants included in the final analysis. Of these, 26 participants (22.8%) were classified as NW, and 88 participants (77.2%) were classified as OW/OB. The proportion of male participants did not differ significantly between the NW and OW/OB groups (61.5% vs. 68.2%, p = 0.53). The mean age of the OW/OB group was significantly lower than that of the NW group (12.3 ± 2.7 vs. 13.5 ± 2.5 years, p = 0.04). The NW group had a higher proportion of participants in the pubertal period compared with the OW/OB group (80.8% vs. 55.7%, p = 0.05). There were no significant differences in caregiver educational level, family income, or urinary creatinine concentrations between the two groups (Table 1).

**Table 1:** Baseline characteristics of study participants according to weight status

**Characteristics**	**All** **(N = 114)**	**Normal weight** **(N = 26)**	**Overweight/obese** **(N = 88)**	**p**
**Age, mean ± SD**	12.6 ± 2.7	13.5 ± 2.5	12.3 ± 2.7	0.04*
**Male, n (%)**	76 (66.7)	16 (61.5)	60 (68.2)	0.53
**Puberty**	70 (61.4)	21 (80.8)	49 (55.7)	0.05
**Weight SDS**	3.40 (1.77–5.97)	0.77 (−0.11–1.32)	4.54 (2.86–6.54)	<0.001*
**Height SDS**	1.62 (0.33–2.88)	1.16 (−0.18–2.51)	2.05 (0.42–3.01)	0.14
**BMI SDS**	2.55 (1.23–3.72)	−0.02 (−0.89–0.34)	3.07 (2.16–4.17)	<0.001*
**Care-giver education level, n (%)**				
**<Bachelor’s degree**	40 (35.1)	5 (19.2)	35 (39.8)	0.078
**Bachelor’s degree**	46 (40.4)	11 (42.3)	35 (39.8)	
**>Bachelor’s degree**	28 (24.6)	10 (38.5)	18 (20.5)	
**Family income per month (Baht), n (%)**				
**<10,000**	6 (5.3)	2 (7.7)	4 (4.6)	0.712
**10,000–25,000**	27 (23.7)	5 (19.2)	22 (25)	
**>25,000**	81 (71.1)	19 (73.1)	62 (70.5)	
**TV hours per day (hours), n (%)**				
**<2 hours**	28 (24.6)	3 (11.5)	25 (28.4)	0.079
**≥2 hours**	86 (75.4)	23 (88.5)	63 (71.6)	

Values are presented as mean ± SD or median (interquartile range), as appropriate. BMI, body mass index; SDS, standard deviation score

### Detection of urinary bisphenols

BPA was detected in all urine samples (100%). BPS was detected in 57 participants (50.0%), with a higher detection rate in the NW group compared with the OW/OB group (65.4% vs. 45.5%); however, this difference did not reach statistical significance (p = 0.07). BPF was detected in 60 participants (53.6%), with no significant difference between the NW and OW/OB groups (50.0% vs. 53.4%, p = 0.76) (Supplement Table 3).

### Body composition

Participants in the OW/OB group had significantly higher body fat mass compared with those in the NW group (median 69.2 kg vs. 48.2 kg, p < 0.001). Skeletal muscle mass was also higher in the OW/OB group (23.4 kg vs. 19.7 kg, p = 0.03). The percentage of body fat in the OW/OB group was approximately twice that observed in the NW group (41.4% vs. 19.7%, p < 0.001). Segmental analysis demonstrated significantly higher fat mass in both arms and the trunk among OW/OB participants. In contrast, leg fat mass did not differ significantly between groups (Table 2).

**Table 2:** Comparison of body composition and metabolic parameters between normal-weight and overweight/obese participants

	**All**	**NW**	**OW/OB**	**p**
**Body composition**				
SMM (kg)	22.6 (16.5–27.8)	19.7 (16.3–24.0)	23.4 (17.1–29.8)	0.03*
PBF (%)	36.7 (29.1–44.0)	19.7 (15.8–26.4)	41.4 (34.8–45.6)	<0.001*
Body fat mass (kg)	62.9 (47.6–83.6)	48.2 (38.6–54.8)	69.2 (54.8–89.2)	<0.001*
Right arm (kg)	2 (1.4–2.6)	1.5 (1.2–2.1)	2.2 (1.4–2.9)	0.001*
Left arm (kg)	1.9 (1.4–2.6)	1.6 (1.1–2)	2.2 (1.5–2.9)	0.002*
Trunk (kg)	18.2 (14–22.5)	15.6 (13.1–18.5)	19.3 (14.4–24.5)	0.005*
Right leg (kg)	6.4 (4.7–8)	5.9 (4.6–7)	6.5 (4.7–8.4)	0.08
Left leg (kg)	6.4 (4.6–8)	5.9 (4.5–7.1)	6.5 (4.7–8.3)	0.08
**Metabolic parameters**				
Fasting plasma glucose (mg/dL)	87 (83–92)	90 (83–96)	87 (83–90)	0.08
HbA1C (%)	5.3 (5.2–5.5)	5.3 (5.2–5.4)	5.4 (5.2–5.5)	0.26
Insulin (µU/mL)	10.6 (7.4–18)	6.3 (5–8.6)	12.9 (8.7–21.4)	<0.001*
HOMA IR	2.2 (1.6–4)	1.6 (1–2.1)	2.7 (1.9–4.5)	<0.001*
HOMA IS	0.5 (0.3–0.6)	0.6 (0.5–1)	0.4 (0.2–0.5)	<0.001*
AST (IU/L)	28 (23–35)	26 (22–30)	28.5 (23.5–36.0)	0.10
ALT (IU/L)	25 (18–38)	19 (15–22)	29 (19–41)	<0.0001*
Total cholesterol (mg/dL)	170 (151–193.5)	162.5 (145–194)	170 (154–193)	0.52
TG (mg/dL)	86 (57–120.5)	62 (44–90)	90 (62–130)	0.003*
LDL (mg/dL)	106 (84–130)	92.5 (80–117)	110 (86–130)	0.08
HDL (mg/dL)	46 (39–54)	54 (46–66)	42.5 (38–52)	<0.001*
Urine Cr (g/L)	0.0013 (0.001–0.002)	0.0012 (0.001–0.002)	0.0013 (0.001–0.002)	0.79

Values are presented as median (interquartile range). ALT, alanine aminotransferase; AST, aspartate aminotransferase; Cr, creatinine; HDL, high-density lipoprotein; HOMA IS, homeostatic model assessment of insulin sensitivity; HOMA IR, homeostatic model assessment of insulin resistance; LDL, low-density lipoprotein; NW, normal weight; OW/OB, overweight/obese; PBF, percent body fat; SMM, skeletal muscle mass; TG, triglyceride

### Metabolic parameters

Fasting insulin levels were significantly higher in the OW/OB group than in the NW group (median 12.9 µIU/mL vs. 6.3 µIU/mL, p < 0.001). Consistent with this finding, the OW/OB group demonstrated significantly higher HOMA-IR values and lower HOMA-IS values, indicating increased insulin resistance (both p < 0.001). Serum ALT levels were significantly higher in the OW/OB group compared with the NW group (p < 0.0001), whereas AST levels did not differ significantly between groups. With regard to lipid profiles, the OW/OB group had significantly higher TG levels (p = 0.003) and lower HDL cholesterol levels (p < 0.001). Total cholesterol and LDL cholesterol levels did not differ significantly between groups (Table 2).

### Creatinine-adjusted urinary bisphenol concentrations

Creatinine-adjusted geometric mean concentrations of BPA did not differ significantly between the OW/OB and NW groups (7.83 vs. 8.32 µg/g creatinine, p = 0.74). Similarly, no significant differences were observed for BPS concentrations between groups (p = 0.75). Although creatinine-adjusted BPF concentrations were higher in the OW/OB group than in the NW group, this difference did not reach statistical significance (0.071 vs. 0.027 µg/g creatinine, p = 0.06). Creatinine-adjusted bisphenol concentrations stratified by quartiles in NW and OW/OB group are shown in Table 3.

**Table 3:** Urinary concentrations of BPA, BPS, and BPF (µg/g creatinine) in normal weight and overweight/obesity group.

	**Total**	**Q1**	**Q2**	**Q3**	**Q4**	**Geometric mean,** **95% CI**
**All** **participants**	**BPA**	1.632–4.523	4.523–7.227	7.227–11.271	11.271–93.231	7.938 (6.830–9.226)
	**BPF**	0.001–0.006	0.006–0.076	0.076–0.463	0.463–10.151	0.057 (0.037–0.087)
	**BPS**	0.002–0.014	0.014–0.087	0.087–0.352	0.352–2.084	0.070 (0.049–0.100)

**NW**	**BPA**	2.071–5.283	5.283–7.790	7.790–8.716	8.716–78.473	8.317^a^ (6.151–11.247)
	**BPF**	0.003–0.004	0.004–0.010	0.01–0.187	0.187–1.364	0.027^b^ (0.011–0.065)
	**BPS**	0.002–0.037	0.037–0.121	0.121–0.242	0.242–1.536	0.078^c^ (0.038–0.161)

**OW/OB**	**BPA**	1.632–4.290	4.290–7.145	7.145–12.781	12.781–93.231	7.829^a^ (6.565–9.337)
	**BPF**	0.001–0.006	0.006–0.167	0.167–0.511	0.511–10.151	0.071^b^ (0.043–0.115)
	**BPS**	0.002–0.013	0.013–0.073	0.073–0.381	0.381–2.084	0.068^c^ (0.045–0.103)

a, b, and c represents *p* value (0.74, 0.06, and 0.75, respectively) for comparisons of the geometric mean of creatinine-adjusted urinary concentrations of BPA, BPS, and BPF between the normal-weight and overweight/obesity groups. BPA, bisphenol A; BPF, bisphenol F; BPS, bisphenol S; Q, quartile; NW, normal weight; OW/OB, overweight/obese

### Associations between bisphenols and metabolic parameters

**Fig. 1:**
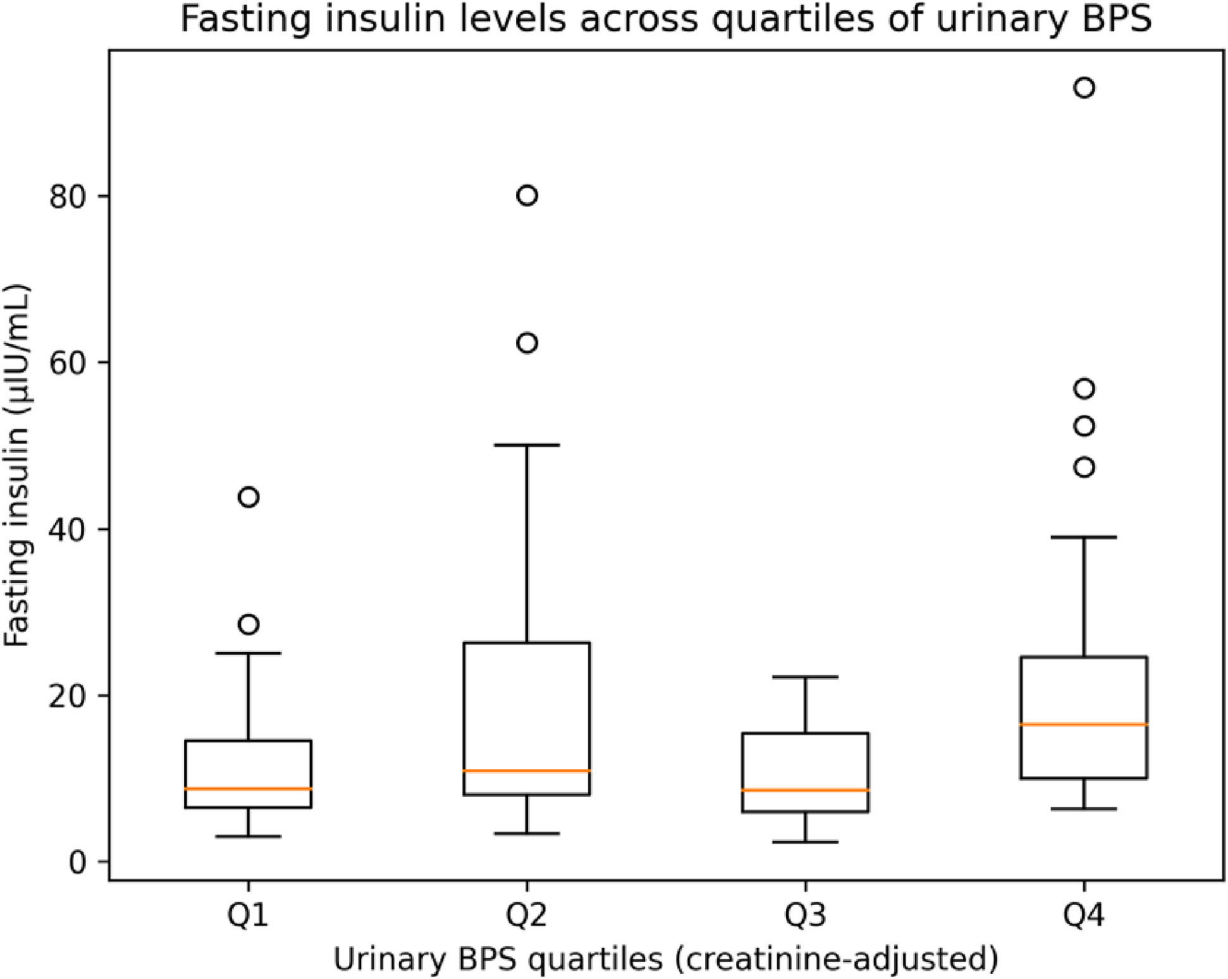
Distribution of fasting insulin levels across quartiles of creatinine-adjusted urinary bisphenol S concentrations. Boxes indicate the interquartile range with median lines, and whiskers represent 1.5 × IQR. Overall differences among quartiles were evaluated using the Kruskal–Wallis’s test, and trends across quartiles were assessed using linear regression. A significant difference was observed between Q4 and Q1 (16.53 vs. 8.74 µU/mL; p = 0.0026). P for trend = 0.125

Urinary BPS concentrations were positively associated with log-transfomed fasting insulin levels in multivariable analysis (aGMR 1.13; 95% CI, 1.05–1.20; p = 0.0001), while higher levels of BPA in the urine were associated with lower insulin levels (aGMR 0.77; 95%CI 0.66–0.90; p = 0.001) (Table 4). The median fasting insulin levels across quartiles of creatinine-adjusted urinary BPS concentrations in Q1, Q2, Q3, and Q4 were 8.74, 10.95, 8.54, and 16.53 µU/mL, respectively (Fig. 1), with significant difference observed between Q4 and Q1 (p = 0.0026). Urine BPA, BPF, and BPS concentration were not significantly associated with obesity status or BMI SDS, HOMA IR, and ALT levels (Supplement Table 4–8). Urinary BPF concentrations showed an association with LDL cholesterol levels (aGMR 5.82; 95%CI 0.52–11.11; p = 0.032) (Supplement Table 8).

**Table 4:** Factors associated with insulin levels

**Factors**	**Univariable**		**Multivariable**	

	**GMR** **(95%CI)**	**P-value**	**aGMR** **(95%CI)**	**P-value**
cr-adjusted urinary BPA (µg/g creatinine)	0.82 (0.69–0.98)	0.030	0.77 (0.66–0.90)	0.001
cr-adjusted urinary BPF (µg/g creatinine)	0.96 (0.90–1.02)	0.200		
cr-adjusted urinary BPS (µg/g creatinine)	1.06 (0.98–1.14)	0.140	1.13 (1.05–1.20)	0.001
Age (yrs)	1.05 (1.00–1.11)	0.040	1.06 (1.01–1.11)	0.011
Obesity (yes/no)	2.04 (1.49–2.80)	<0.001	2.07 (1.55–2.77)	<0.001
Puberty (yes/no)	1.36 (1.02–1.82)	0.040		
Body fat mass (kg)	1.02 (1.01–1.02)	<0.001		
SMM (kg)	1.05 (1.03–1.06)	<0.001		
PBF (%)	1.02 (1.01–1.03)	<0.001		
ALT (IU/L)	1.01 (1.00–1.01)	<0.001	1.01 (1.00–1.01)	0.001
TG (mg/dL)	1.00 (1.00–1.01)	<0.001		
LDL (mg/dL)	1.00 (1.00–1.00)	0.820		

Cr, creatinine; SMM, skeletal muscle mass; PBF, percent body fat; ALT, alanine aminotransferase; TG, triglyceride; LDL, low-density lipoprotein; HDL, high-density lipoprotein

## Discussion

In this cross-sectional study of Thai children and adolescents, we examined the associations between urinary concentrations of BPA, BPS, and BPF and obesity-related metabolic parameters. The principal findings were that (1) urinary concentrations of BPA, BPS, and BPF did not differ according to weight status and (2) higher urinary BPS concentrations were independently associated with higher fasting insulin levels; in contrast, higher urinary BPA concentration were associated with lower fasting insulin levels, and (3) higher urinary BPF concentration were associated with higher LDL cholesterol levels in Thai children. Together, these findings suggest that BPA substitutes, particularly BPS and BPF, may have metabolic relevance in children even in the absence of overt differences in adiposity.

Previous epidemiological studies investigating the relationship between BPA exposure and childhood obesity across different populations and age groups have reported inconsistent results. While several studies have demonstrated positive associations between urinary BPA concentrations and body mass index or waist circumference, others have failed to identify such relationships especially for BPS and BPF. In U.S. adults aged ≥20 years, Liu et al. (2017) [26] found that higher BPA exposure was significantly associated with general obesity (OR 1.78; 95% CI: 1.10–2.89; p = 0.04), whereas no significant associations were observed for BPF or BPS, suggesting that these analogues may not be strongly related to obesity in adult populations at current exposure levels. In pediatric populations, however, stronger associations have been reported. Jacobson et al. demonstrated that urinary BPS concentrations were positively associated with both general obesity (OR 1.16; 95% CI: 1.02–1.32) and abdominal obesity (OR 1.13; 95% CI: 1.02–1.27) among children and adolescents aged 6–19 years [8]. Detectable urinary BPF levels were also linked to a higher prevalence of abdominal obesity (OR 1.29; 95% CI: 1.01–1.64) and increased BMI z-scores, whereas BPA and total bisphenols were not significantly associated with obesity outcomes in this younger group [8]. Similarly, Liu et al. (2019), in a cohort of 745 participants aged 6–17 years, reported elevated odds of general obesity among those in the highest quartile of urinary bisphenol concentrations compared with the lowest quartile. The adjusted ORs were 1.74 for BPA, 1.54 for BPF, and 1.36 for BPS after controlling for demographic, socioeconomic, and lifestyle factors [6]. Collectively, these findings suggest that bisphenol analogues, particularly BPF and BPS, may exert more pronounced obesity-related effects in pediatric populations than in adults. In the present study, urinary BPA concentrations were comparable between normal-weight and overweight/obese participants. One possible explanation is the ubiquitous nature of BPA exposure, as BPA was detected in all urine samples, which may have limited the contrast across BMI categories. Moreover, metabolic alterations such as insulin resistance may precede measurable changes in body composition, particularly in pediatric populations.

An important finding of this study is the observed association between urinary BPS concentrations and fasting insulin levels. Although the magnitude of this association was modest, small effect sizes are commonly observed in studies of widespread environmental exposures and may have population-level relevance. Hyperinsulinemia is an early marker of insulin resistance and represents an important precursor of type 2 diabetes mellitus in both children and adults if left unaddressed. Evidence directly linking BPS exposure to hyperinsulinemia remains limited, and available findings have been inconsistent with our results. Nevertheless, several epidemiological studies have reported significant associations between urinary BPS concentrations and the risk of type 2 diabetes. Moreno-Gómez-Toledano et al. reported a strong relationship between urinary BPS, but not BPF, and diabetes risk. Individuals in the highest exposure quartile (Q4) showed significantly increased odds of diabetes, particularly among women (OR 1.94; 95% CI: 1.61–2.35) [27]. Similarly, Rancière et al. demonstrated that the association between BPS exposure and incident diabetes appeared stronger in women [HR 4.23 (95% CI: 1.69–10.63)] than in men [HR 1.76 (95% CI: 0.93–3.33)], with suggestive evidence of effect modification by sex (p for interaction = 0.09) [28]. These findings indicate that the metabolic effects of BPS may vary according to sex and other host factors such as age, BMI, and family history of diabetes. We also found that children with bisphenol concentrations in the highest quartile had significantly higher insulin levels compared with those in the lower quartiles, potentially reflecting a threshold association with hyperinsulinemia. Taken together, our observation of higher fasting insulin levels among children with greater BPS exposure may reflect early metabolic disruption that could precede the high HOMA IR, obesity, insulin resistance, and overt type 2 diabetes later in life. Although HOMA-IR is a widely used and validated surrogate marker of insulin resistance, fasting insulin may serve as a more sensitive indicator in the early stages of insulin resistance, when fasting glucose levels remain within the normal range. Experimental evidence suggests that BPS may disrupt insulin homeostasis by interfering with insulin signaling pathways and impairing pancreatic β-cell function through estrogen receptor–mediated mechanisms. Marroqui et al. reported that both short- and long-term exposure to BPS and BPF can enhance glucose-stimulated insulin secretion, a response that may contribute to the development of type 2 diabetes [29]. The acute effects appear to involve inhibition of ATP-sensitive potassium (KATP) channels, whereas chronic exposure may exert more complex actions, including modulation of voltage-gated Ca^2+^, Na^+^, and K^+^ ion channels. Together, these mechanistic and epidemiological findings support the biological plausibility that BPS exposure may contribute to early metabolic disturbances, indicating that this BPA substitute is not metabolically inert.

The observed association between higher BPA concentrations and lower fasting insulin levels aligns with experimental models suggesting that BPA may exert direct cytotoxic effects on pancreatic beta-cells [30]. Unlike BPS, which appears to promote peripheral insulin resistance, BPA has been linked to impaired glucose-stimulated insulin secretion in the pancreas, reflecting a state of secretory impairment rather than metabolic compensation [30].

BPF exposure was associated with higher LDL cholesterol levels in our cohort. While studies on BPF in children are scarce, adult data have linked both BPA and BPF to dyslipidemia, specifically low HDL-C and elevated triglycerides [25]. Notably, while previous meta-analyses of US children (NHANES 2003–2014) found no significant lipid disruptions associated with BPA [31], our results highlight BPF as a potentially more potent lipid disruptor in the pediatric population.

### Strengths and limitations

Several limitations of this study should be acknowledged. First, the observational and cross-sectional design precludes causal inference; thus, our findings cannot establish a definitive causal or reverse-causal relationship. Second, bisphenol exposure was assessed using single spot urine samples, which may not reflect long-term exposure as bisphenol concentrations may vary over time; however, this approach is widely used and considered acceptable for ranking exposure in population-based studies. Third, the relatively small sample size, particularly in the normal-weight group, may have limited statistical power to detect modest associations. Finally, residual confounding related to dietary patterns and other environmental exposures cannot be entirely excluded. Despite these limitations, this study has several strengths. We simultaneously measured BPA and its major substitutes using sensitive LC–MS/MS methods and comprehensively assessed metabolic parameters and body composition in a pediatric population. Importantly, data on BPA substitutes in children from Southeast Asia remain scarce, and our findings contribute novel regional evidence to the existing literature.

## Conclusions

Although urinary concentrations of BPA, BPS, and BPF did not differ by weight status, higher urinary BPS and BPF concentrations were associated with hyperinsulinemia and high LDL cholesterol levels in Thai children and adolescents, respectively. These findings suggest that BPA substitutes, particularly BPS and BPF, may have metabolic effects and warrant further investigation from an environmental health and preventive medicine perspective. Longitudinal studies are needed to clarify the temporal relationships and potential causal pathways underlying these associations.

## Supplementary information

**Additional file 1:** **Supplement table 1.** The intra-day, inter-day and %recovery of BPA, BPS and BPF at LQC, MQC and HQC concentration levels. **Supplement table 2.** The chromatogram of blank matrix and spiked 5 ng/uL BPA, BPS and BPF. **Supplement Table 3.** Detection rates of BPA, BPS, and BPF in the urine samples. **Supplement table 4.** Factors associated with obesity status (yes / no). **Supplement table 5.** Factors associated with BMI SDS. **Supplement table 6.** Factors associated with HOMA IR. **Supplement table 7.** Factors associated with LDL levels. **Supplement table 8.** Factors associated with ALT levels.

## Declarations

### Availability of data and material

All data are incorporated into the article and its online supplementary material.

### Data availability statement

The supplementary tables supporting the findings of this study are provided in the online repository, and the de-identified summary data are available upon request.
