# Supplementary material for: BPS and BPF linked to metabolic alterations independent of adiposity in Thai children and adolescents
Source: Environ Health Prev Med. 2026 Jun 4;31:37. doi: 10.1265/ehpm.26-00027 (PMC13254261; doi:10.1265/ehpm.26-00027)
Supplement: Supplementary file 1 — Additional file 1: Supplement table 1. The intra-day, inter-day and %recovery of BPA, BPS and BPF at LQC, MQC and HQC concentration levels. Supplement table 2. The chromatogram of blank matrix and spiked 5 ng/uL BPA, BPS and BPF. Supplement Table 3. Detection rates of BPA, BPS, and BPF in the urine samples. Supplement table 4. Factors associated with obesity status (yes / no). Supplement table 5. Factors associated with BMI SDS. Supplement table 6. Factors associated with HOMA IR. Supplement table 7. Factors associated with LDL levels. Supplement table 8. Factors associated with ALT levels. [file ehpm-31-037-s001.docx]

**Supplement table 1.** The intra-day, inter-day and %recovery of BPA, BPS and BPF at LQC, MQC and HQC concentration levels.

|  | | **BPA**  **concentrations (ng/uL)** | **%Recovery** | **Intra-day** | **Inter-day** |
| --- | --- | --- | --- | --- | --- |
|  |  |  |  | **%Accuracy (%CV)** | **%Accuracy (%CV)** |
| Urine | LQC | 1.5 | 96.75 | 97.12 (3.08) | 103.63 (5.45) |
|  | MQC | 30 | 96.10 | 96.54 (3.58) | 93.33 (3.54) |
|  | HQC | 70 | 89.15 | 94.75 (1.99) | 92.79 (7.93) |

|  | | BPS  concentrations (ng/uL) | % recovery | Intra-day | Inter-day |
| --- | --- | --- | --- | --- | --- |
|  |  |  |  | %Accuracy (%CV) | %Accuracy (%CV) |
| Urine | LQC | 0.03 | 88.75 | 98.00 (4.56) | 101.83 (3.29) |
|  | MQC | 30 | 102.74 | 85.50 (0.26) | 88.29 (4.28) |
|  | HQC | 70 | 94.92 | 87.96 (1.96) | 90.69 (2.71) |

|  | | BPF  concentrations (ng/uL) | % recovery | Intra-day | Inter-day |
| --- | --- | --- | --- | --- | --- |
|  |  |  |  | %Accuracy (%CV) | %Accuracy (%CV) |
| Urine | LQC | 0.03 | 98.38 | 99.33 (12.42) | 106.00 (7.86) |
|  | MQC | 30 | 112.02 | 85.45 (4.95) | 89.08 (3.53) |
|  | HQC | 70 | 101.55 | 85.56 (2.46) | 88.82 (3.73) |

LQC (Low quality control), MQC (Medium quality control) and HQC (High quality control)

**Supplement table 2.** The chromatogram of blank matrix and spiked 5 ng/uL BPA, BPS and BPF

| Name | Blank | STD 5 ng/uL |
| --- | --- | --- |
| BPA (Urine) | 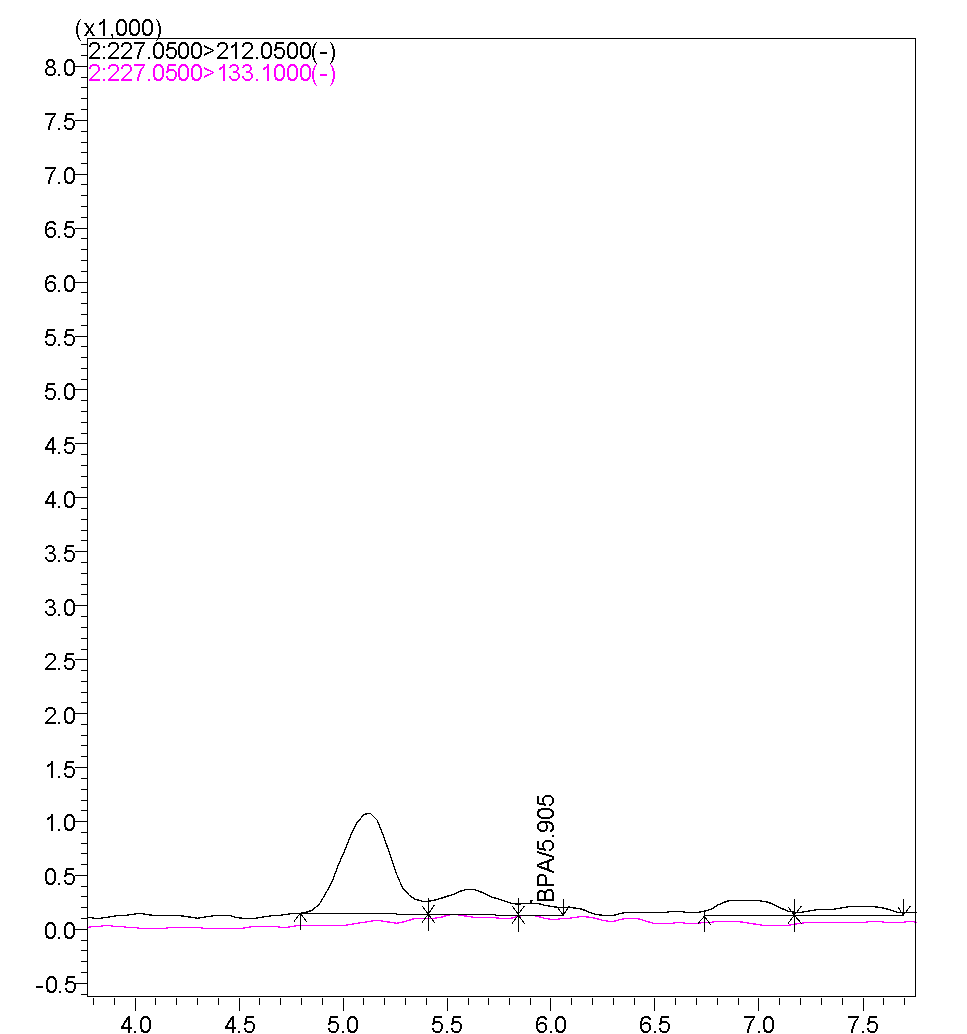 | 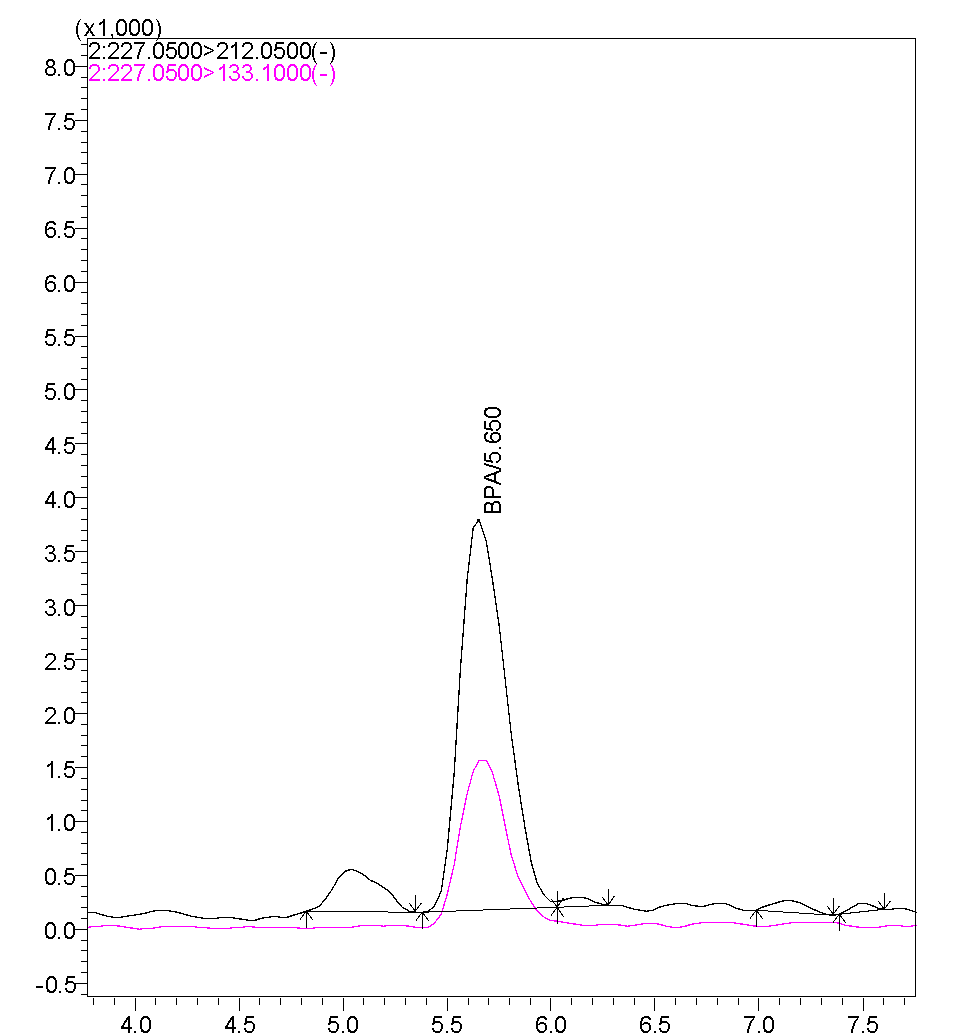 |
| BPS (Urine) | 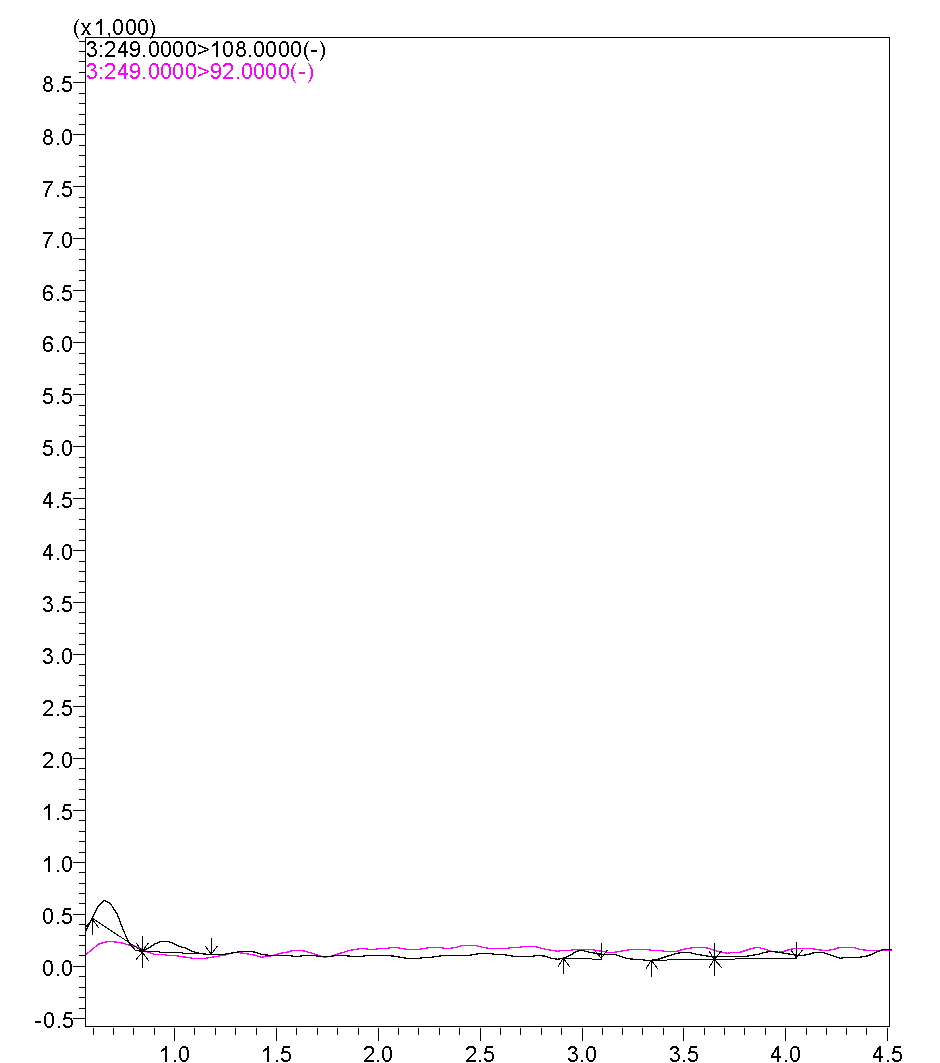 | 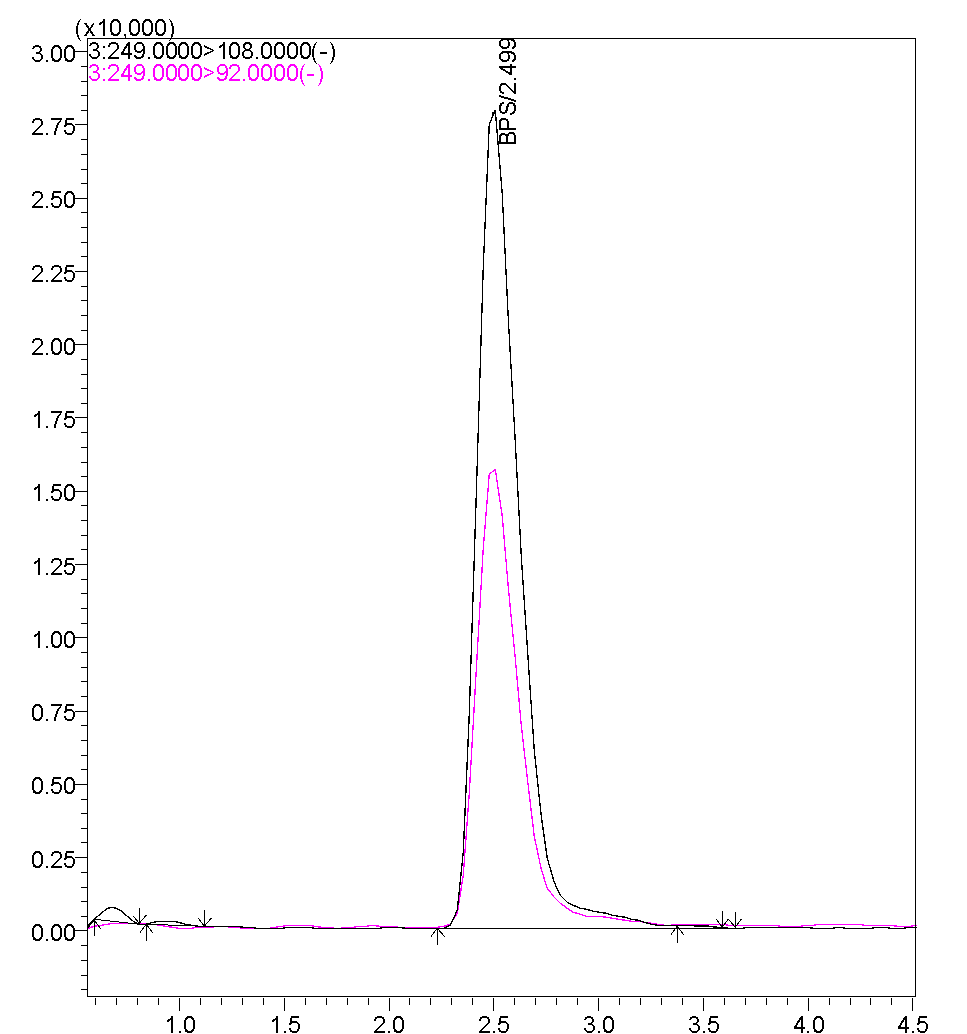 |
| BPF (Urine) | 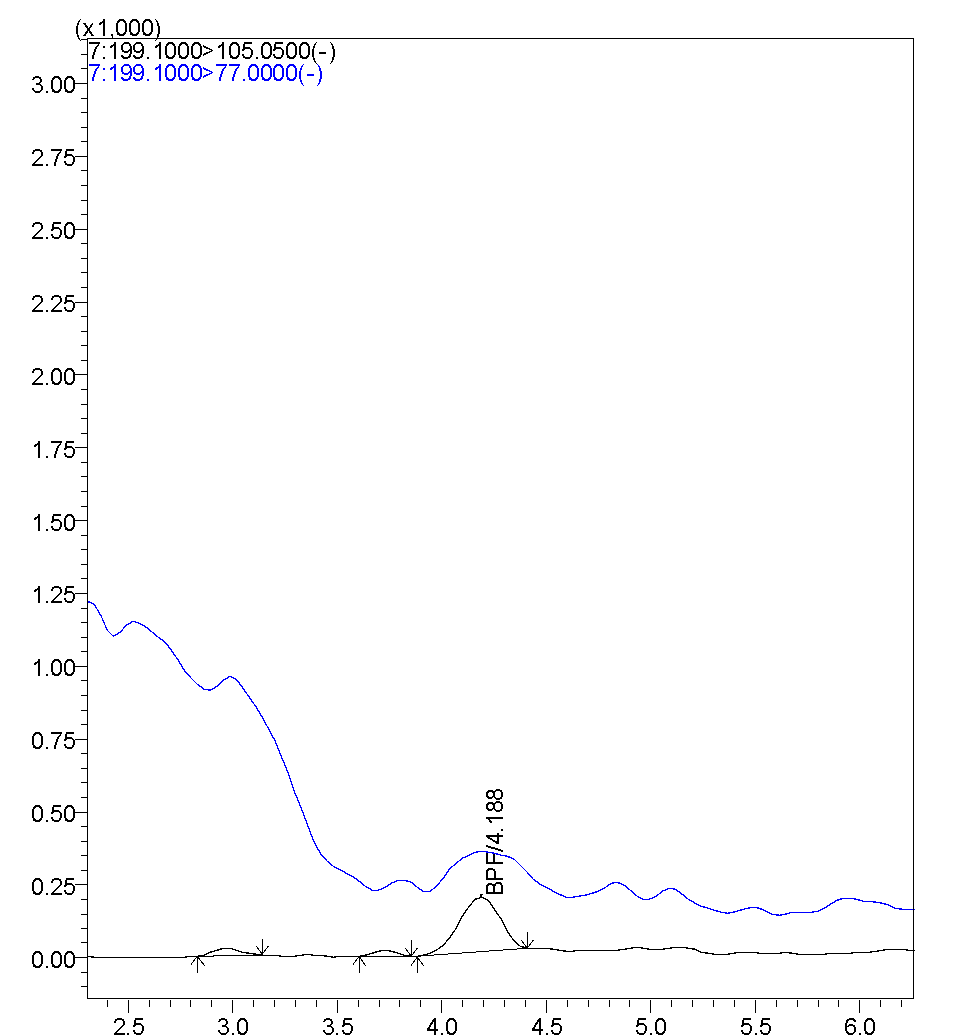 | 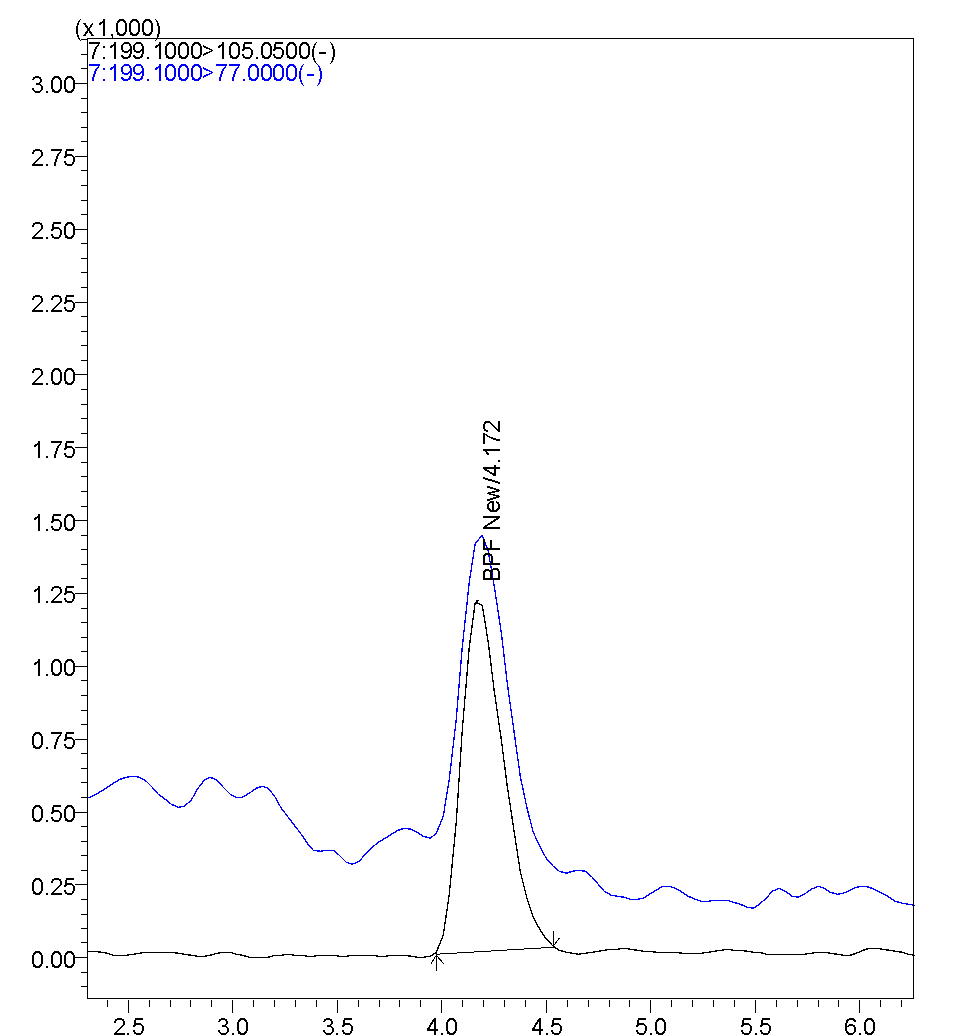 |

BPA, bisphenol A; BPF, bisphenol F; BPS, bisphenol S

**Supplement Table 3.** Detection rates of BPA, BPS, and BPF in the urine samples

| **Percent detection Urinary** | **BMI SDS** | | | **P-value** |
| --- | --- | --- | --- | --- |
|  | **All**  **(N=114)** | **Normal weight**  **(N=26)** | **Overweight/obese**  **(N=88)** |  |
| **BPA, N (%)** | 114 (100) | 26 (100) | 88 (100) | NA |
| **BPS, N (%)** | 57 (50) | 17 (65.4) | 40 (45.5) | 0.07 |
| **BPF, N (%)** | 60 (53.6) | 13 (50) | 47 (53.4) | 0.76 |

Detection defined as concentration above the limit of detection. BPA, bisphenol A; BPF, bisphenol F; BPS, bisphenol S

**Supplement table 4.** Factors associated with obesity status (yes / no)

| **Factors** | **Univariable** | | **Multivariable** | |
| --- | --- | --- | --- | --- |
|  | **OR**  **(95%CI)** | **P-value** | **aOR**  **(95%CI)** | **P-value** |
| Cr-adjusted urinary BPA (µg/g creatinine) | 1.00 (0.97 to 1.03) | 0.924 |  |  |
| Cr-adjusted urinary BPF (µg/g creatinine) | 2.57 (0.63 to 10.55) | 0.191 |  |  |
| Cr-adjusted urinary BPS (µg/g creatinine) | 1.32 (0.40 to 4.43) | 0.648 |  |  |
| Age (years) | 0.83 (0.70-0.99) | 0.041 | 0.48 (0.24-0.98) | 0.044 |
| Body fat mass (kg) | 1.08 (1.04 to 1.12) | <0.001 |  |  |
| SMM (kg) | 1.07 (1.01 to 1.13) | 0.029 | 1.19 (1.11-2.01) | 0.009 |
| PBF (%) | 1.33 (1.19 to 1.50) | <0.001 | 1.41 (1.02-1.69) | <0.00 |
| Puberty (yes) | 0.35 (0.12 to 1.03) | 0.057 |  |  |
| Fasting plasma glucose (mg/dL) | 0.98 (0.95 to 1.03) | 0.463 |  |  |
| Insulin (μIU/mL) | 1.22 (1.08 to 1.38) | 0.002 |  |  |
| HOMA IR | 2.32 (1.34 to 4.01) | 0.003 |  |  |
| ALT (IU/L) | 1.03 (1.00 to 1.06) | 0.064 |  |  |
| LDL (mg/dL) | 1.01 (1.00 to 1.03) | 0.170 |  |  |

OR, odd ratio; aOR, adjusted-odd ratio; Cr, creatinine; SMM, skeletal muscle mass; PBF, percent body fat; HOMA IR, homeostatic model assessment of insulin resistance; ALT, alanine aminotransferase; LDL, low-density lipoprotein

**Supplement table 5.** Factors associated with BMI SDS

| **Factors** | **Univariable** | | **Multivariable** | |
| --- | --- | --- | --- | --- |
|  | **Coefficient**  **(95%CI)** | **P-value** | **Adjusted coefficient**  **(95%CI)** | **P-value** |
| Cr-adjusted urinary BPA (µg/g creatinine) | -0.01 (-0.04 to 0.02) | 0.391 |  |  |
| Cr-adjusted urinary BPF (µg/g creatinine) | 0.11 (-0.23 to 0.45) | 0.504 |  |  |
| Cr-adjusted urinary BPS (µg/g creatinine) | -0.09 (-1.03 to 0.84) | 0.844 |  |  |
| Age (years) | -0.17 (-0.30 to -0.04) | 0.012 | -0.38 (-0.44 to -0.32) | <0.001 |
| Body fat mass (kg) | 0.05 (0.04 to 0.06) | <0.001 |  |  |
| SMM (kg) | 0.08 (0.04 to 0.12) | <0.001 | 0.16 (0.15 to 0.18) | <0.001 |
| PBF (%) | 0.13 (0.11 to 0.15) | <0.001 | 0.11 (0.10 to 0.12) | <0.001 |
| Puberty (yes) | -0.98 (-1.74 to -0.22) | 0.012 | -0.33 (-0.65 to -0.004) | 0.048 |
| Fasting plasma glucose (mg/dL) | -0.01 (-0.05 to 0.03) | 0.527 |  |  |
| Insulin (μIU/mL) | 0.05 (0.02 to 0.07) | <0.001 |  |  |
| HOMA IR | 0.15 (0.06 to 0.23) | 0.001 |  |  |
| ALT (IU/L) | 0.02 (0.01 to 0.03) | 0.001 |  |  |
| LDL (mg/dL) | 0.00 (-0.01 to 0.02) | 0.403 |  |  |

GMR, geometric mean ratio; aGMR, adjusted-geometric mean ratio; Cr, creatinine; SMM, skeletal muscle mass; PBF, percent body fat; HOMA IR, homeostatic model assessment of insulin resistance; ALT, alanine aminotransferase; LDL, low-density lipoprotein

**Supplement table 6.** Factors associated with HOMA IR

| **Factors** | **Univariable** | | **Multivariable** | |
| --- | --- | --- | --- | --- |
|  | **Coefficient**  **(95%CI)** | **P-value** | **Adjusted coefficient**  **(95%CI)** | **P-value** |
| Cr-adjusted urinary BPA (µg/g creatinine) | -0.04 (-0.11 to 0.02) | 0.163 |  |  |
| Cr-adjusted urinary BPF (µg/g creatinine) | -0.22 (-0.93 to 0.48) | 0.529 |  |  |
| Cr-adjusted urinary BPS (µg/g creatinine) | 0.69 (-1.55 to 2.94) | 0.541 |  |  |
| Age (years) | 0.22 (-0.07 to 0.50) | 0.134 |  |  |
| Obesity (yes) | 2.50 (0.66 to 4.35) | 0.008 |  |  |
| BMI SDS | 0.65 (0.27 to 1.04) | 0.001 |  |  |
| Body fat mass (kg) | 0.07 (0.05 to 0.10) | <0.001 |  |  |
| SMM (kg) | 0.20 (0.12 to 0.29) | <0.001 |  |  |
| PBF (%) | 0.05 (-0.01 to 0.12) | 0.108 |  |  |
| Puberty (yes) | 1.56 (-0.09 to 3.21) | 0.063 |  |  |
| Fasting plasma glucose (mg/dL) | 0.23 (0.162 to 0.288) | <0.001 | 0.08 (0.06 to 0.09) | <0.001 |
| Insulin levels (μIU/mL) | 0.26 (0.25 to 0.27) | <0.001 | 0.24 (0.23 to 0.25) | <0.001 |
| ALT (IU/L) | 0.05 (0.02 to 0.07) | <0.001 | 0.005 (0.001 to 0.009) | 0.024 |
| LDL (mg/dL) | 0.00 (-0.01 to 0.02) | 0.403 |  |  |

GMR, geometric mean ratio; aGMR, adjusted-geometric mean ratio; Cr, creatinine; SMM, skeletal muscle mass; PBF, percent body fat; HOMA IR, homeostatic model assessment of insulin resistance; ALT, alanine aminotransferase; LDL, low-density lipoprotein

**Supplement table 7.** Factors associated with LDL levels

| **Factors** | **Univariable** | | **Multivariable** | |
| --- | --- | --- | --- | --- |
|  | **Coefficient**  **(95%CI)** | **P-value** | **Adjusted coefficient**  **(95%CI)** | **P-value** |
| Cr-adjusted urinary BPA (µg/g creatinine) | 0.11 (-0.32 to 0.54) | 0.624 |  |  |
| Cr-adjusted urinary BPF (µg/g creatinine) | 5.61 (0.18 to 11.05) | 0.043 | 5.82 (0.52 to 11.11) | 0.032 |
| Cr-adjusted urinary BPS (µg/g creatinine) | -7.31 (-23.76 to 9.14) | 0.380 |  |  |
| Age (years) | 0.12 (-2.11 to 2.36) | 0.913 |  |  |
| Obesity (yes/no) | 9.85 (-4.26 to 23.97) | 0.169 |  |  |
| BMI SDS | 1.30 (-1.77 to 4.36) | 0.403 |  |  |
| Body fat mass (kg) | 0.02 (-0.21 to 0.26) | 0.849 |  |  |
| SMM (kg) | 0.07 (-0.64 to 0.78) | 0.844 |  |  |
| PBF (%) | 0.10 (-0.41 to 0.62) | 0.697 |  |  |
| Puberty (yes) | -2.48 (-15.74 to 10.78) | 0.712 |  |  |
| Fasting plasma glucose (mg/dL) | 0.34 (-0.27 to 0.94) | 0.273 |  |  |
| Insulin levels (μIU/mL) | -0.07 (-0.47 to 0.33) | 0.742 |  |  |
| HOMA IR | 0.14 (-1.38 to 1.65) | 0.857 |  |  |
| ALT (IU/L) | 0.23 (0.05 to 0.41) | 0.014 | 0.23 (0.06 to 0.41) | 0.011 |

GMR, geometric mean ratio; aGMR, adjusted-geometric mean ratio; Cr, creatinine; SMM, skeletal muscle mass; PBF, percent body fat; HOMA IR, homeostatic model assessment of insulin resistance; ALT, alanine aminotransferase; LDL, low-density lipoprotein

**Supplement table 8.** Factors associated with ALT levels

| **Factors** | **Univariable** | | **Multivariable** | |
| --- | --- | --- | --- | --- |
|  | **Coefficient**  **(95%CI)** | **P-value** | **Adjusted coefficient**  **(95%CI)** | **P-value** |
| Cr-adjusted urinary BPA (µg/g creatinine) | -0.14 (-0.58 to 0.30) | 0.539 |  |  |
| Cr-adjusted urinary BPF (µg/g creatinine) | -0.82 (-6.46 to 4.83) | 0.775 |  |  |
| Cr-adjusted urinary BPS (µg/g creatinine) | -6.40 (-21.91 to 9.11) | 0.415 |  |  |
| Age (years) | 2.13 (-0.08 to 4.34) | 0.058 |  |  |
| Obesity (yes/no) | 13.90 (-0.37 to 28.17) | 0.056 |  |  |
| BMI SDS | 5.31 (2.34 to 8.28) | 0.001 |  |  |
| Body fat mass (kg) | 0.53 (0.32 to 0.75) | <0.000 | 0.33 (0.49 to 1.58) | 0.008 |
| SMM (kg) | 0.99 (0.31 to 1.68) | 0.005 |  |  |
| PBF (%) | 0.89 (0.40 to 1.39) | 0.001 | 0.66 (0.14 to 1.18) | 0.013 |
| Puberty (yes) | 7.27 (-5.09 to 19.63) | 0.246 |  |  |
| Fasting plasma glucose (mg/dL) | 1.10 (0.52 to 1.68) | <0.000 | 1.03 (0.49 to 1.58) | <0.00 |
| Insulin levels (μIU/mL) | 0.67 (0.28 to 1.07) | 0.001 |  |  |
| HOMA IR | 3.00 (1.55 to 4.46) | <0.000 |  |  |
| LDL (mg/dL) | 0.24 (0.05 to 0.43) | 0.014 | 0.20 (0.03 to 0.37) | 0.021 |

GMR, geometric mean ratio; aGMR, adjusted-geometric mean ratio; Cr, creatinine; SMM, skeletal muscle mass; PBF, percent body fat; HOMA IR, homeostatic model assessment of insulin resistance; ALT, alanine aminotransferase; LDL, low-density lipoprotein
